# Structure and Function Insight of the α-Glucosidase QsGH13 From *Qipengyuania seohaensis* sp. SW-135

**DOI:** 10.3389/fmicb.2022.849585

**Published:** 2022-03-03

**Authors:** Xingyu Zhai, Kaijuan Wu, Rui Ji, Yiming Zhao, Jianhong Lu, Zheng Yu, Xuewei Xu, Jing Huang

**Affiliations:** ^1^Department of Parasitology, School of Basic Medical Science, Central South University, Changsha, China; ^2^Department of Microbiology, Xiangya School of Medicine, Central South University, Changsha, China; ^3^State Key Laboratory of Genetic Engineering, School of Life Sciences, Fudan University, Shanghai, China; ^4^Key Laboratory of Marine Ecosystem Dynamics, Second Institute of Oceanography, Ministry of Natural Resources, Hangzhou, China

**Keywords:** QsGH13, α-glucosidase, characterization, β → α loop, crystal structure

## Abstract

The α-glucosidases play indispensable roles in the metabolic mechanism of organism, prevention, and treatment of the disease, and sugar hydrolysis, and are widely used in chemical synthesis, clinical diagnosis, and other fields. However, improving their catalytic efficiency and production to meet commercial demand remains a huge challenge. Here we detected a novel GH13 family α-glucosidase, QsGH13, from the deep-sea bacterium *Qipengyuania seohaensis* sp. SW-135. QsGH13 is highly substrate specific and only hydrolyzes sugars containing alpha-1,4 glucoside bonds. For example, its enzymatic activity for *p*-nitrophenyl-α-D-glucopyranoside was 25.41 U/mg, and the *K*_m_ value was 0.2952 ± 0.0322 mM. The biochemical results showed that the optimum temperature of QsGH13 is 45°C, the optimum pH is 10.0, and it has excellent biological characteristics such as alkali resistance and salt resistance. The crystal structure of QsGH13 was resolved with a resolution of 2.2 Å, where QsGH13 is composed of a typical TIM barrel catalytic domain A, a loop-rich domain B, and a conserved domain C. QsGH13 crystal belonged to the monoclinic space group P2_1_2_1_2_1_, with unit-cell parameters *a* = 58.816 Å, *b* = 129.920 Å, *c* = 161.307 Å, α = γ = β = 90°, which contains two monomers per asymmetric unit. The β → α loop 4 of QsGH13 was located above catalytic pocket. Typical catalytic triad residues Glu202, Asp266, and Glu329 were found in QsGH13. The biochemical properties and structural analysis of QsGH13 have greatly improved our understanding of the catalytic mechanism of GH13 family. This study provides new ideas to broaden the application of α-glucosidase in alcohol fermentation, glycolysis, and other industries.

## Introduction

The CAZy database^1^ classifies proteins into glycoside hydrolases (GHs), glycosyl transferases, polysaccharide lyases, carbohydrate esterases, auxiliary activities, and carbohydrate-binding modules based on amino acid sequence similarity and spatial structure of their catalytic structural domains (Henrissat, 1991; Cantarel et al., 2009). The GH13 family belongs to the GHs members and is mainly composed of (β/α)_8_-barrel fold catalytic domain A, a loop-rich domain B, and a conserved domain C (Banner et al., 1975). The enzymes of the GH13 family catalyze reactions mainly through a double-displacement mechanism, in which catalytic nucleophilic (Asp) and acid/base catalyst (Glu) play an essential role (McCarter and Withers, 1994).

The α-glucosidases (EC:3.2.1.20) are members of GHs and are widely distributed in all organisms that are specific mainly for the exohydrolysis of α-1,4-glycosidic linkages at the non-reducing end of polysaccharides, such as maltooligosaccharides, and release α-D-glucose. Alternatively, it combines free glucose residues with the α-1,4-glycosidic linkages in oligosaccharides to form α-1,6-glycosidic linkages, and obtain non-fermentative oligosaccharides (McCarter and Withers, 1994; Hondoh et al., 2008). The α-glucosidases play key roles in clinical detection, prevention, and treatment of the disease, metabolic mechanism of the organism, and alcohol fermentation, sugar hydrolysis, widely used in chemical fields such as chemical synthesis. Most commercially used α-glucosidases are produced by fungi, but they all have certain limitations, such as moderate thermostability, high acidic pH requirements, many byproducts, and slow catalytic activity, which greatly increase production cost. Therefore, it is urgent to find novel α-glucosidase to improve glucose production and catalytic efficiency.

In this study, the gene *qsgh13* encoding α-glucosidase (QsGH13) was identified from the marine bacteria *Qipengyuania seohaensis* sp. SW-135 (*Qs*. SW-135) and heterologously expressed in soluble form in *Escherichia coli* BL21(DE3) plus. Biochemical results showed that QsGH13 has strong substrate specificity and excellent biological properties such as alkali and salt resistance. Structural analysis showed that QsGH13 has a typical TIM barrel catalytic domain A, the loop-rich domain B, and the conserved domain C. The β → α loop 4 of QsGH13, located above the catalytic pocket was significantly different from other enzymes in GH13, suggesting that it is related to substrate specificity (Noguchi et al., 2003). Investigation into the biochemical characteristics and structure of QsGH13 might greatly improve our understanding of the molecular mechanism of the GH13 family and further indicate their physiological and biochemical basis for adapting to extreme conditions, thus, improving the industrial application value of α-glucosidase.

## Materials and Methods

### Bioinformatic Analysis

The amino acid sequences with the first 100 similarities to QsGH13 sequences were obtained using NCBI BLASTp.^2^ Clustal was used for amino acid multiple sequence alignment of QsGH13 and its homologs^3^ (Larkin et al., 2007). Secondary structure alignment results were obtained using ESpript based on multiple sequence alignment results and QsGH13 spatial coordinates^4^ (Robert and Gouet, 2014). The maximum likelihood estimation (MLE) in MEGA X was used to construct the phylogenetic tree (Kumar et al., 2018).

### Cloning, Expression, and Purification of Recombinant Proteins

*Qs.* SW-135 (basionym: *Erythrobacter seohaensis* sp. SW-135) (Taxonomy ID: 266951, GenBank Accession: GCA_002795865.1) (Kwon et al., 2007; Xu et al., 2020; Supplementary Figure 1), belonging to the family Erythrobacteraceae, was isolated from deep-sea sediment samples that were collected from the East Pacific Seamount region. The QsGH13-encoding gene (*qsgh13*) was identified and cloned from the genome of *Qs.* SW-135. To be specific, the *qsgh13* gene was amplified by PCR (forward: 5′-GGCGGATCCATGAGCGGCAAGCTGCCTTG-3′, *Bam*HI, reverse: 5′-GCGGAGCTCTCATGTGTCGGTCTCCAGGATGA-3′, *Sac*I) and inserted into the expression vector pSMT3 (Li et al., 2012) using the *Bam*HI and *Sac*I restriction sites.

The recombinant *E. coli* BL21(DE3) plus/pSMT3-*qsgh13* was cultured in LB medium containing 50 μg/ml of kanamycin and 34 μg/ml of chloramphenicol at 37°C. When the OD_600_ reached 0.8, the recombinant protein expression was induced by 0.5 mM isopropyl-β-D-thiogalactopyranoside (IPTG) for 20 h at 16°C (Shen et al., 2019). Cells were harvested by centrifugation at 5,000 rpm for 15 min at 4°C. The cell precipitation was resuspended in a lysis buffer [50 mM Tris, pH 8.0; 500 mM NaCl; 1% (v/v) glycerol; 10 mM imidazole; 1 mM β-Me; 0.2 mM PMSF] and was disrupted on ice with an ultrasonic crusher (SCIENIZ, Ningbo, China). The separated supernatant was purified by Ni-NTA affinity chromatography. After the recombinant protein with 6xHis-Sumo tag was digested with Ulp1 enzyme, the target protein was obtained in the flow-through fractions and was concentrated with a 50-kDa concentrator. Then QsGH13 protein was purified in a buffer (20 mM Tris, pH 7.4; 100 mM NaCl; 2 mM DTT) by gel-filtration chromatography (Superdex 200 16/600, GE, United States). The protein was determined by SDS-PAGE, and the concentration was measured by the method of Bradford with bovine serum albumin (BSA) as a standard (Huang et al., 2016).

### Functional Characterization

The enzymatic activity and substrate specificity of wild-type QsGH13 were determined by p-nitrophenol method (Irma et al., 1992). The 100-μl standard reaction buffer consisted of 1 mM *p*NPαGlu, 20 mM Gly-NaOH buffer (pH 10.0), and an appropriate amount of purified enzyme. The enzymatic activity was determined by continuously measuring the amount of released *p*-nitrophenol at the absorbance of 405 nm using Multiskan FC (ThermoFisher, MA, United States), at 45°C, and the inactivated enzyme solution was used as the blank control. All results were carried out in three independent experiments. The absorbance of 405 nm was measured every 2 min, and the total reaction time was 30 min.

The substrate specificity of the enzyme was determined using *p*-nitrophenyI-β-D-glucopyranoside (*p*NPβGlu), *p*-nitrophenyl-α-D-glucopyranoside (*p*NPαGlu), *p*NP-α-L-arabinopyranoside (*p*NPαArap), *p*-nitrophenyl -β-D-lactopyranoside (*p*NPβLac), *p*-nitrophenyI-α-D-galacto pyranoside (*p*NPαGal), *p*-nitrophenyI-β-D-galactopyranoside (*p*NPβGal), *p*-nitrophenyI-β-D-mannopyranoside (*p*NPβ Man), *p*-nitrophenyI-β-D-xylopyranoside (*p*NPβXyl), and *p*-nitrophenyI-β-D-cellobioside (*p*NPβCel) as substrates, respectively. All the substrates were purchased from Shanghai Yuanye Bio-Technology Co., Ltd.

The kinetic parameters were examined using *p*NPαGlu as a substrate at different concentrations varying from 0.025 to 2.0 mM. The Michaelis–Menten constant (*K*_m_) and maximum velocity (*V*_max_) were analyzed by the Michaelis–Menten equation using GraphPad Software (GraphPad prism5, United States) (Huang et al., 2019).

The optimum temperature of the enzyme was determined with different temperatures varying from 4 to 70°C. Buffers with different pH were used to determine the optimal pH of the enzyme, including 20 mM citrate buffer (pH 3.0–pH 6.5), 20 mM phosphate buffer (pH 6.5–pH 7.5), 20 mM Tris–HCl buffer (pH 7.5–pH 9.0), and 20 mM Gly-NaOH buffer (pH 9.0–pH 13.0).

The influence of metal ions on the enzyme activity was determined by adding 10 mM of cations (Ba^2+^, Ca^2+^, Co^2+^, Cu^2+^, K^+^, Fe^2+^, Fe^3+^, Mg^2+^, Mn^2+^, Na^+^, Ni^2+^, Sr^2+^, Zn^2+^) and ethylene diamine tetraacetic acid (EDTA) to the standard reaction buffer. The effect of organic solvents on enzyme activity was investigated in the presence of 10% (v/v) acetonitrile, acetone, dimethyl sulfoxide, ethanol, formic acid, isopropanol, methanol, respectively. The influences of the detergents on enzyme activity were examined by using 10% (v/v) Triton X-100, Triton X-114, Tween 20, Tween 80, and SDS. The enzyme activity assay was carried out under optimal standard reaction buffer with 20 mM Gly-NaOH buffer (pH 10.0) at 45°C, and the enzyme activity in the blank group was defined as 100% without additives.

The salt tolerance of enzyme was measured in the presence of 1.0, 2.0, 3.0, 4.0, or 5.0 M NaCl, and the enzyme activity in the blank group without NaCl was defined as 100%. The tolerance of enzyme on products were tested by adding 0.5, 1.0, 1.5, 2.0, 2.5, and 3.0 M glucose to 20 mM Gly-NaOH buffer (pH 10.0). In the absence of the substrate, the thermostability of the enzyme was determined by measuring the residual activity at the optimum conditions after incubating the enzyme for different periods of time at 4°C, respectively. Similarly, in the absence of the substrate, the alkali resistance of enzyme was measured by testing the residual activity at the optimum conditions after incubating the enzyme for 2, 24 h in buffers of different pH at 4°C.

### High-Performance Liquid Chromatography

The enzymatic activities of QsGH13 toward maltose, sucrose, isomaltose, panose, and isommaltose were determined by HPLC. First, glucose, maltose, isomaltose, panose, and isommaltose trisaccharide were dissolved in mobile phase to establish the standard curve. Second, the reaction buffer contained 15% substrate (w/v), 1 mg/ml of QsGH13, and 20 mM Gly-NaOH buffer (pH 10.0), which were used to catalyze the reaction at 45°C. Meanwhile, QsGH13 in the reaction buffer was replaced by the deactivation of QsGH13, which served as a negative control. The reaction of the samples were terminated by high-temperature denaturation, and the supernatant was collected by centrifugation at 12,000 rpm for 10 min. The samples were detected by Thermo Scientific*™* Hypersil*™* APS-2 HPLC with 72% acetonitrile and 28% water as mobile phase. Glucose concentration was calculated according to the standard curve.

### Data Collection for Crystallographic Structure Determination

The purified QsGH13 was concentrated to 10 mg ml^–1^ using Amicon Ultra 50K ultrafiltration devices (Merck Millipore, Darmstadt, Germany). The crystallization of QsGH13 was prepared by handing drop vapor diffusion methods by mixing 1 μl of QsGH13 (10 mg ml^–1^) with an equal volume of reservoir solution at 4°C. It was grown in a condition of 0.1 M Tris–HCl (pH 8.5), 0.2 M NaCl, and 20% PEG 3,350. The crystals were briefly soaked with the cryoprotectant solution, and then flash cooled directly in liquid nitrogen at −173°C. The x-ray diffraction data were collected at the BL17U beamline of SSRF (Shanghai Synchrotron Radiation Facility, Shanghai, China). The data were processed and scaled using *XDS* (Kabsch, 2010). The collected data and processing details are shown in Table 1.

**TABLE 1:** Data collection and refinement statistics of QsGH13.

Items	QsGH13
**Data collection**	
Wavelength	0.9793
Resolution range (Å)	50.00–2.20 (2.80–2.20)
Space group	P2_1_2_1_2_1_
a, b, c (Å)	*a* = 58.816, *b* = 129.920, *c* = 161.307
α, γ, β (°)	α = γ = β = 90.00
Total reflections	841,069
Unique reflections	121,073
Completeness (%)	99.40 (97.70)
R_merge_	11.5 (24.6)
I/σ (I)	14.36 (6.55)
CC_1/2_	99.3 (93.2)
**Refinement statistics**	
Resolutions (Å)	34.28–2.20 (2.33–2.20)
Completeness (%)	99.54
Reflection used in refinement	63,429 (6,188)
Final R_work_	0.2112 (0.2252)
Final R_free_	0.2678 (0.2785)
**No. of atoms**	
Water	771
Protein residues	1,036
Total	9,014
**RMSD**	
Bond lengths (Å)	0.013
Angles (°)	1.620
Average B-factor (Å^2^)	23.95
**Ramachandran plot**	
Ramachandran favored (%)	95.41
Ramachandran allowed (%)	4.10
Ramachandran outliers (%)	0.49
Rotamer outliers (%)	1.30
**PDB code**	7VOH

The structure of QsGH13 was determined by molecular replacement method with Phaser (McCoy et al., 2007), using the crystal structure of α-glucosyltransferase XgtA from *Xanthomonas campestris* WU-9701 (PDB entry: 6AAV, 51% identity to QsGH13) (Watanabe et al., 2020) as a search model. The refinement data of QsGH13 was performed using REFMAC and Phenix (Liebschner et al., 2019). Coot was used to further modify and adjust the structure (Emsley and Cowtan, 2004). The refinement statistics data of QsGH13 are shown in Table 1.

### Structure Visualization

The disordered area of QsGH13 sequence was predicted using IUPred2A.^5^ The catalytic pocket of QsGH13 was predicted using POCASA.^6^ The visualization of the tunnel in the QsGH13 structure used CAVER 3.0 PyMol plugin. The docking model between QsGH13 and its substrate was predicted with AutoDock 4 (Morris et al., 2009). All of the 3D structures were analyzed and drawn using PyMOL program (Version 2.0 Schrödinger, LLC).

## Results

### Cloning, Expression, and Purification of QsGH13

**FIGURE 1:**
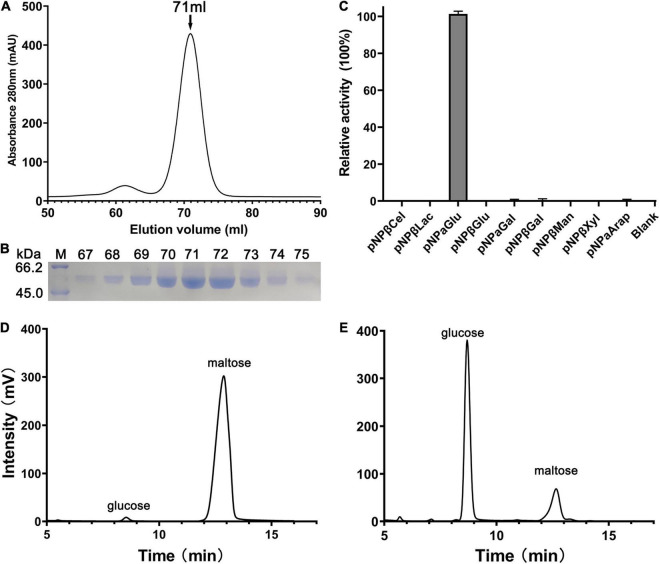
Purification and substrate specificity of QsGH13. **(A)** The QsGH13 protein was purified by gel-filtration chromatography, and it was eluted at the peak of 71 ml. **(B)** The QsGH13 protein was determined by SDS-PAGE. **(C)** The substrate specificity of the enzyme was determined using different substrates. **(D,E)** The enzymatic activities of QsGH13 toward maltose was determined by HPLC. Maltose (substrate) and glucose (product) came out at the peak positions of 12.723 and 8.831 min, respectively. Panel **(D)** is before the reaction, and Panel **(E)** is after the reaction.

Nucleotide sequence analysis showed that the open reading frame of 1,587 bp *qsgh13* gene sequence from deep-sea sediment metagenomic screening encodes a protein of 528 amino acids with a theoretical molecular weight of 59.3 kD. Phylogenetic tree sequence analysis based on NCBI, PDB, and CAZy databases showed that the protein QsGH13 belongs to the GH13 family (Supplementary Figure 1), and predicted that the protein QsGH13 had α-glucosidase characteristics. To explore the physicochemical characterizations of protein QsGH13, we constructed the gene *qsgh13* into the recombinant plasmid with an N-terminal His_6_-SUMO label in *E. coli* DH5α and expressed the protein QsGH13 in *E. coli* BL21 (DE3) plus. Cells were cultured and enriched, and protein His_6_-SUMO-QsGH13 was extracted. After the His_6_-SUMO tag was removed by Ulp1 enzyme, the protein QsGH13 was purified by gel-filtration chromatography to 95% homogeneity. Pure QsGH13 (12.6 ± 2.9 mg) was obtained from 1-L cultures. SDS-PAGE was used to show the molecular weight of QsGH13 (Figures 1A,B). QsGH13 was found to specifically hydrolyze *p*-nitrophenyl-α-D-glucopyranoside (*p*NPαGlu), but no other substrates (Figure 1C). The enzymatic activities of QsGH13 toward maltose was determined by HPLC. Maltose (substrate) and glucose (product) came out at the peak positions of 12.68 and 8.69 min, respectively (Figures 1D,E and Supplementary Figure 2). Therefore, QsGH13 was identified as α-glucosidase.

### Biochemical Characterization of QsGH13

**FIGURE 2:**
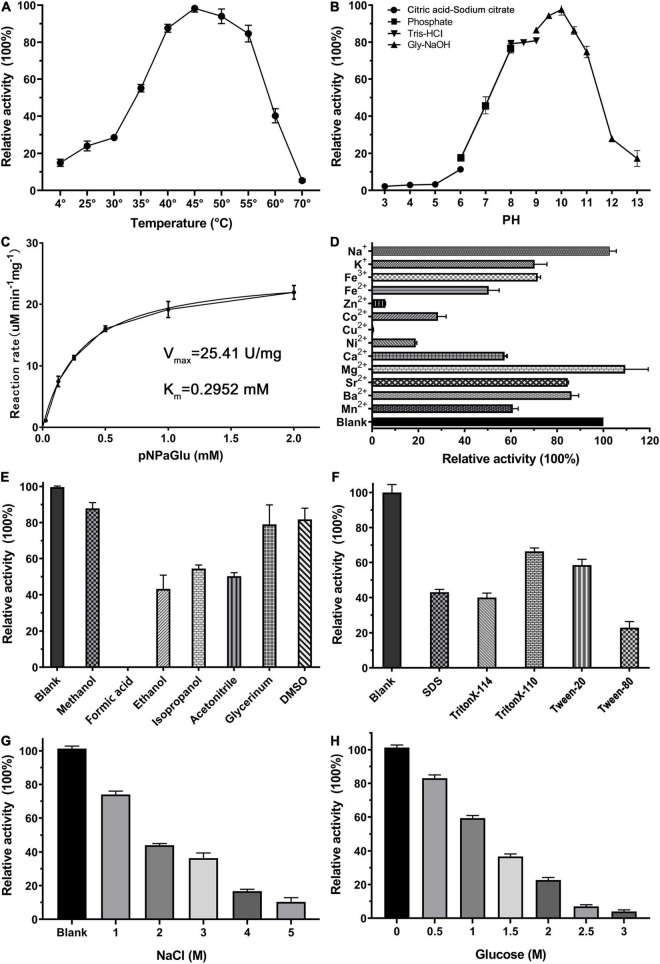
Biochemical characterization of QsGH13. **(A,B)** The effect of temperature and pH on enzyme activity of QsGH13. The value obtained at 45°C and pH 10.0 was taken as 100%, respectively. **(C)** The specific activity of QsGH13. **(D–H)** Effects of different metal ions (10 mM), detergents [10% (v/v)], organic solvents [10% (v/v)], NaCl concentration, and glucose concentration on the enzymatic activity. The values of blank control were taken as 100%.

We have studied the optimum reaction conditions of QsGH13 toward *p*NPαGlu. QsGH13 was able to maintain 80% catalytic activity toward *p*NPαGlu at a temperature range of 40–55°C and a pH range of 8.0–11.0; QsGH13 showed the highest catalytic activity at 45°C and pH 10.0 (Figures 2A,B). The catalytic activity of QsGH13 was almost unchanged when stored at 4°C for 24 h (Supplementary Figure 3A). The catalytic activity of QsGH13 was decreased with the increase in incubation time in different pH buffers (Supplementary Figure 3B). The enzymatic activity of QsGH13 was 25.41 U/mg, and the *K*_m_ value was 0.2952 ± 0.0322 mM (Figure 2C).

Furthermore, we investigated the tolerance ability of QsGH13 to the addition of different cations, detergents, organic solvents, and different concentrations of NaCl and glucose added into the reaction buffer under optimum temperature and pH conditions (Figures 2D–H). The α-glucoside activity of QsGH13 was completely eliminated with the addition of Cu^2+^, and only about 10–30% was retained with Zn^2+^, Ni^2+^, Co^2+^, and 50–80% activity remained with K^+^, Fe^2+^, Fe^3+^, and the activity of QsGH13 reached more than 100% with Mg^2+^, Na^+^. Moreover, the α-glucosidase activity of QsGH13 was comparative with the blank under the addition of 10% (v/v) organic solvents (methanol, formic acid, ethanol, isopropanol, acetonitrile, glycerol, and DMSO) or 10% (v/v) detergent (SDS Triton X-T114, Triton X-100, Tween20, and Tween80); it was attenuated. Especially, it is completely inactivated after the addition of formic acid (Figures 2E,F). In addition, the α-glucosidase activity of QsGH13 was decreased with the increase in NaCl concentration. The α-glucosidase activity of QsGH13 was less than 40% of the blank group under the addition of 4 M NaCl (Figure 2G). QsGH13 still retained 30% activity compared with the blank group under the condition of 2 M glucose, but with an increase in glucose concentration, its activity was gradually abolished (Figure 2H).

### The Overall Structure of QsGH13

**FIGURE 3:**
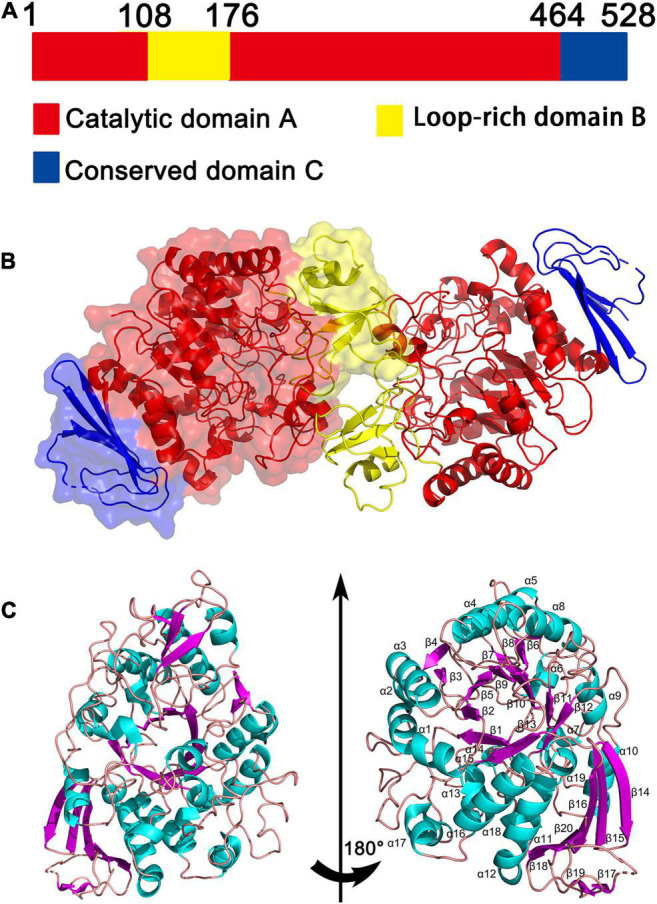
The overall structure of QsGH13. **(A)** QsGH13 is composed of the catalytic domain A (red), loop-rich domain B (yellow), and conserved domain C (blue). **(B)** Schematic of the overall structure of the CmGH1 protein. **(C)** Cartoon representation of QsGH13 with the labeled secondary structure elements. α-Helices were shown in magenta; β-strands were denoted as lake blue arrows.

The crystal structures of QsGH13 molecules were fully built to 2.2-Å resolution with the satisfied *R*_work_ and *R*_free_ values of 21.83 and 27.38%, respectively. According to the diffraction dataset, the QsGH13 crystal belongs to the monoclinic space group P2_1_2_1_2_1_, with unit-cell parameters *a* = 58.815 Å, *b* = 129.920 Å, *c* = 161.304 Å, α = γ = β = 90°, which contains two monomers per asymmetric unit (Table 1). Phylogenetic tree analysis showed that QsGH13 belonged to α-glucosidase (EC 3.2.1.20), GH13 family, subfamily 23 (Supplementary Figure 1). QsGH13 has typical and conserved characteristics of the GH13 family (Figure 3A), where the catalytic domain A (residues 1–107, 176–463) is composed of a typical TIM barrel, sandwiched between the loop-rich domain B and the conserved domain C, and it is mainly composed of (β/α) eight-barrel structures, including eight α-helical and eight β-fold. The loop-rich domain B (residues 108–175) is derived from the domain A, including an α-helix and three β-fold, which are involved in the formation of QsGH13 dimers and forms catalytic pockets with domain A. Domain C (residues 464–525) is composed of multiple highly conserved β-folds that stabilizes the entire protein structure (Shen et al., 2015; Figures 3B,C). In order to achieve catalytic activity, dimers formed by two monomers are essential in the GH13 family of enzymes (Watanabe et al., 2020; Wangpaiboon et al., 2021). The QsGH13 monomers formed dimers mainly through five hydrogen bonds and 105 non-bonded contacts (Supplementary Figure 4). After the superposition of two monomers, the root-mean-square deviation (r.m.s.d.) value was 0.250 Å using the DALI server^7^ (Holm, 2020). It shows that the two chains are very similar in structure and has no obvious biological significance, so we only study one monomer.

**FIGURE 4:**
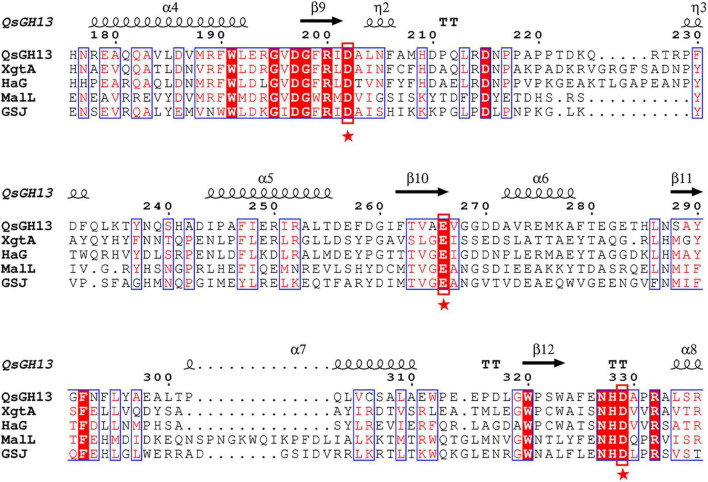
Multiple sequence alignment of QsGH13 and other members of the GH13 family. The sequences include alpha-glucosyl transfer enzyme XgtA from *Xanthomonas campestris* (PDB ID:6AAV), alpha-glucosidase HaG from *Halomonas* sp. H11 (PDB ID:3WY1), oligo-1,6-glucosidase MaIL from *Bacillus subtilis* subsp. subtilis (PDB ID:4M56), and alpha-glucosidase GSJ from *Geobacillus* sp. HTA-462 (PDB ID: 2ZE0). The conserved catalytic residues in the GH13 family were marked with a red box. The asterisk represents the catalytic residue.

Multiple sequence alignments showed that QsGH13 had high homology with other members of the GH13 family, such as XgtA (PDB entry: 6AAV), an α-glucosyl transfer enzyme derived from *Xanthomonas* campestris WU-9701 (Watanabe et al., 2020); HaG (PDB entry: 3WY1), an α-glucosidase produced from *Halomonas* sp. H11 (Shen et al., 2014); GSJ (PDB entry: 2ZE0), an α-glucosidase from *Geobacillus* sp. strain HTA-42 (Shirai et al., 2008); and MalL (PDB entry: 4m8u), an oligo-1,6-glucosidase from *bacillus subtilus* (Hobbs et al., 2013; Supplementary Table 1). The similarity of amino acid sequence from high to low is 51.3, 47.8, 39.6, and 37.5% (Figure 4).

### The β → α Loop 4 of QsGH13

**FIGURE 5:**
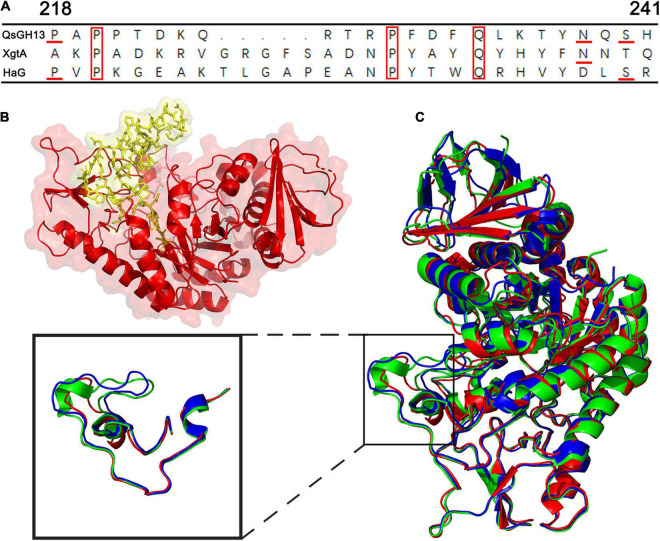
The β → α loop 4 of QsGH13 in comparison with other proteins. **(A)** Superposition of amino acid residues of QsGH13 with amino acid residues of other proteins in the β → α loop 4; the red box represents the conserved amino acid residue. **(B)** Sticks represent the β → α loop 4 of QsGH13. **(C)** The monomer structure comparison of QsGH13 (red), XgtA (green), and HaG (blue). The blue box represents the β → α loop 4 domain of the protein. The β → α loop 4 domain of the protein; the black box represents the different structures of the three proteins.

The overall structures of these GH13 families were similar through superimposing the overall structures of QsGH13 on XgtA, HAG, and GSJ, as indicated by r.m.s.d. values between Cαs in these three structures and the QsGH13 (0.680, 0.722, and 1.426 Å, respectively). However, as shown in Figure 5A, their β-α loop 4 regions were significantly different. The β → α loop 4 of QsGH13 is located above the catalytic pocket (Figure 5B), and it is associated with substrate specificity. It controls substrate entry by maintaining structural integrity around the catalytic pocket, and its amino acid sequence is poorly conserved. The β → α loop 4 of QsGH13 has some differences in composition and structure compared with the other three proteins. Amino acid sequence analysis showed that QsGH13 has the highest similarity with XgtA (51.3%); however, the amino acid sequence at 203–242 of QsGH13 just shows a homology of 17.4% (4/23 amino acids are identical) with that of XgtA and a homology of 21.7% with that of HaG. The size of β → α loop 4 of QsGH13 is between GSJ and HaG (Figure 5C). The β → α loop 4 of QsGH13 is demonstrated to be more dynamic than other regions of QsGH13 and the β → α loop 4 of HaG and XgtA by IUPred2A analysis^8^ (Erdõs and Dosztányi, 2020). Furthermore, the more dynamic β → α loop 4 of QsGH13 may be involved in the acquisition and recognition of regulatory substrates (Supplementary Figure 5). The structural differences between QsGH13, XgtA, HaG, and GSJ at the β → α loop 4 region may, therefore, result in the differences observed for the formation of byproducts during α-glucosylation reaction.

### The Catalytic Pocket of QsGH13

**FIGURE 6:**
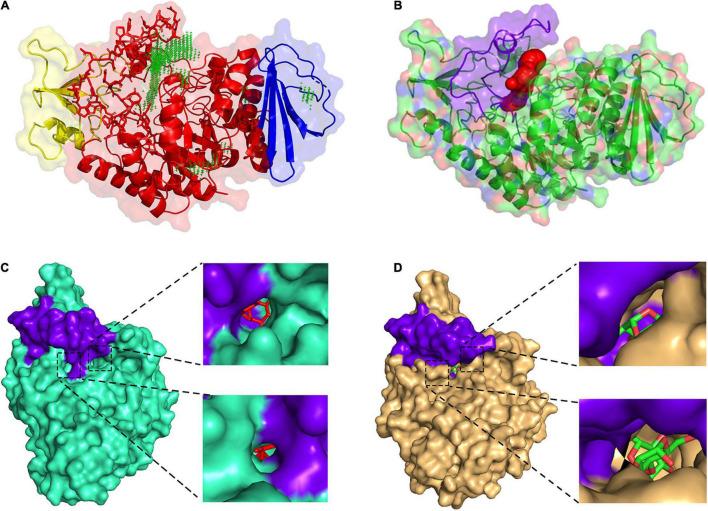
Comparison of the catalytic pocket of QsGH13 and HaG. **(A)** QsGH13 catalytic pocket prediction result; green represents catalytic pocket. **(B)** The shape and size of the QsGH13 catalytic pocket. **(C)** The schematic diagram of the catalytic pocket of HaG. **(D)** The schematic diagram of the catalytic pocket of QsGH13. Purple represents the β → α loop 4 region; on the left is the overall schematic diagram of the protein catalytic pocket, the upper right is the right view of the catalytic pocket, and the lower right is the left view of the catalytic pocket.

The predicted results of the QSGH13 catalytic pocket are consistent with the structure comparison results, and the channel volume is 672 Å^3^, and the average VD is 3.70238 (Figures 6A,B). As shown in Figure 6C, the β → α loop 4 of HaG covers most of the entrance of the catalytic pocket, hinders the entry of long-chain substrates, and almost only hydrolyzes disaccharides (Figure 6C). Compared with HaG, the entrance region of the catalytic site of QsGH13 is relatively large, and the two tunnels are connected to the active center, but the channel is relatively narrow, and the larger substrate cannot pass through (Figure 6D).

**FIGURE 7:**
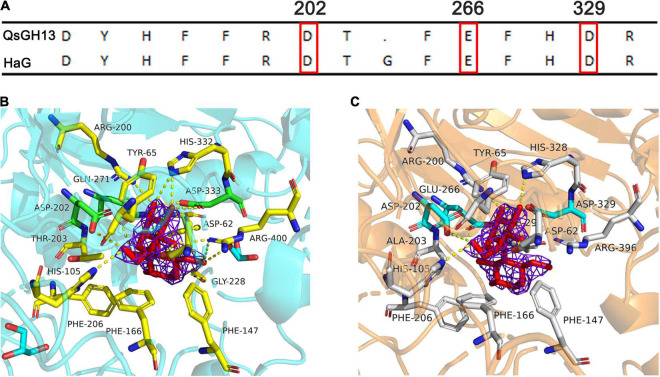
Comparison of QsGH13 and HaG of amino acid residues near the active site. **(A)** Alignment of amino acid residues near the substrate recognition site. The red box indicates the catalytic site. **(B)** Fifteen amino acid residues around the catalytic sites of HaG. **(C)** Fourteen amino acid residues around the catalytic sites of QsGH13. Dashed lines indicate hydrogen bonding with the enzyme and substrate. The purple network represents the electron density cloud.

QsGH13 forms a deep and narrow catalytic pocket of 14 residues in (β/α) eight-barrel structures (TIM) away from the dimeric interface. Among these residues, there are eight residues, including Tyr65, His105, Arg200, Glu266, Asp202, Phe292, His328, and Asp329, that are conserved in the GH13 family. Six residues, Asp62, Phe147, Phe166, Thr203, Phe206, and Arg396, are conserved between QsGH13 and HaG (Figure 7A). The enzymatic hydrolysis of glycosidic bond takes place *via* general acid catalysis that requires two critical residues. In the catalytic pocket of HaG, Asp202, Glu271 (3wy4-Gln271), and Asp333 constitute the catalytic site, of which Asp202 is the nucleophilic residue, Glu271 is the proton acceptor/donor, and Asp333 helps to stabilize Glu271 residue (Figure 7B). Similarly, the structure of QsGH13-maltose formed by docking shows not only how maltose is bound prior to hydrolysis but also the roles of Glu266 as a proton donor for interglycosidic oxygen of maltose and Asp202 as a catalytic nucleophile (Figure 7C). The distance between the proton acceptor/donor (Glu266) and the catalytic nucleophile (Asp202) is 6 Å, and similar conditions have been observed in other retaining glycosidases (Davies and Henrissat, 1995).

## Discussion

The commercial demands for α-glucosidases are so great that even small increments in catalytic efficiency and production might provide enormous commercial value. Traditional genetic engineering techniques have largely solved the production problem, but the main challenge remains to improve its process performance to a practical level. Thus, the key in identifying highly efficient and enhanced α-glucosidases lies in screening for microorganisms and molecules in different environments or engineering existing enzymes. α-Glucosidase, derived from extreme environmental microbes, has some unique physical and chemical functions. For example, marine-derived α-glucosidases usually have excellent properties associated with the marine environment, such as temperature stability, salt tolerance, and alkali tolerance (Sala et al., 2021). Currently, most commercialized α-glucosidases have a hydrolytic amylase activity of 1,000–30,000 U and a transglycosidase activity of 30–5,000 U (Supplementary Table 2; Yamamoto et al., 2004; Naested et al., 2006; Wang et al., 2009; Chen et al., 2010), such as *Aspergillus* of the Japanese AMANO and *Aspergillus oryzae* of Novozymes A/S, both produced by fungi and have an acidic optimum pH and a high optimum temperature. However, deep-sea bacteria-derived QsGH13 reached the highest catalytic activity at 45°C and pH 10.0, which showed high tolerance to salt and alkali. This is related to the evolutionary difference of *Qs*. SW-135, which is an adaptive selection for extreme deep-sea environments. The thermal stability of α-glucosidase may be positively correlated with the number of hydrophobic amino acids, such as glycine and alanine, which may be due to hydrophobic forces that lead to tighter enzyme structures (Krohn and Lindsay, 1993). Mg^2+^ and Na^+^ ions can increase the relative activity of QsGH13, and it is likely due to Mg^2+^ and Na^+^ ions directly coordinating with the functional group of the transition state, which, in turn, stabilizes the space structure and charge in the transition state to enhance enzyme activity (Gohara and Di Cera, 2016), or they can bind to a site not indirectly contacting with the substrate and improve catalytic activity through conformational transitions (Gan et al., 2002; Wheatley et al., 2015; Gohara and Di Cera, 2016).

Compared with other α-glucosidases, QsGH13 has extremely high substrate specificity, which reduces byproducts and improves catalytic efficiency. This excellent characteristic may be related to the conformation of the β → α loop 4 region and the volume of the catalytic pocket. For example, the β → α loop 4 of HaG covers most of the entrance of the catalytic pocket, hinders the entry of long-chain substrates, and almost only hydrolyzes disaccharides (Shen et al., 2014). The β → α loop 4 of XgtA is similar in length to that of HaG, and the channel is relatively narrow and occupied by water molecules that are strongly held by surface amino acids, thus, blocking the entry of hydrophobic substances, such as L-menthol (Watanabe et al., 2020), while α-glucosidase GSJ from *Geobacillus* sp., which has bigger catalytic pocket, can utilize a variety of substrates, even quite large molecules, such as curcumin (Shirai et al., 2008). The size of the β → α loop 4 of QsGH13 is between GSJ and HaG. The entrance region of the catalytic site of QsGH13 is large, with two tunnels connected to the active center, but due to the relatively narrow channel, the larger substrate cannot pass through, so only *p*NPαGlu and maltose can be hydrolyzed by QsGH13. Unlike GSJ, which is able to produce oligosaccharides as a byproduct, QsGH13, XgtA, and HAG hardly ever produce oligosaccharides because they have a long β → α loop 4, which hinders the acceptor-maltose from entering the center of the catalytic pocket, leading to an impossible α-glycosylation reaction (Shirai et al., 2008; Shen et al., 2014; Watanabe et al., 2020). Moreover, compared with other conserved domains, the β → α loop 4 has a larger structural flexibility and reduces the energy potential of the conformation change (Supplementary Figure 5), so it is easier to realize the change in various conformations; thus, it participates in the acquisition and recognition of regulatory substrates. Unlike HaG, which can hydrolyze maltose and sucrose, QsGH13 just hydrolyzes maltose, not sucrose. Although there is enough space for sucrose to successfully enter the substrate catalytic pocket and bind to the recognition sites, sucrose cannot be hydrolyzed because its fructosyl group fails to be recognized due to the substitution of amino acid residues (Shen et al., 2014). These structural differences may lead to differences in product formation.

Compared with other α-glucosidases, QsGH13 has higher catalytic activity (25.41 U/mg), which may be related to its unique catalytic mechanism. Most enzymes of the family GH13 perform catalytic reactions through a two-step double-displacement mechanism, including the formation and decomposition of a covalent glycosyl-enzyme intermediate. Both steps are performed through an oxocarbenium ion transition state (McIntosh et al., 1996; Ravaud et al., 2007; Shen et al., 2015). The structure of QsGH13-maltose formed by docking shows not only how maltose is bound prior to hydrolysis but also the roles of Glu266 as a proton donor for interglycosidic oxygen of maltose and Asp202 as a catalytic nucleophile. First, QsGH13 recognizes substrates specifically by β → α loop 4, allowing maltose to enter the catalytic pocket and binding to residue Asp202 specifically, assisted by residue Asp329. Since the near-linear interaction between maltose O-1 and Glu266 would minimize the proton transfer energy barrier in the catalytic process, subsequently, the catalytic nucleophile Asp202 attacks the C1 of maltose to displace the aglycon, and form an oxocarbenium ion transition state and a molecule of glucose. Meanwhile, the proton receptor/donor Glu266 protonates the glycosidic oxygen as the bond cleaves. Finally, QsGH13 is hydrolyzed by water, with the residue Glu266 deprotonating the water molecule as water attacks. The QsGH13 catalytic pocket is relatively large, with a volume of about 672 Å^3^. When the substrate occupies the pocket, the activity center of QsGH13 provides water continuously and efficiently while releasing the product (Supplementary Figure 6). This process greatly improves catalytic efficiency (Robert et al., 2003). Research has shown that the substrate specificity and the modes of action of these enzymes are controlled by exquisite details of their 3D structures rather than by their overall structures (Davies and Henrissat, 1995). Although it is not clear how many amino acids are involved in the catalytic process of QsGH13, we found that eight residues in the catalytic pocket, including Tyr65, His105, Arg200, Glu266, Asp202, Phe292, His328, and Asp329, are conserved in the family of GH13. Six residues Asp62, Phe147, Phe166, Thr203, Phe206, and Arg396 are conserved in the homologs.

In this study, α-glucosidase (QsGH13) was screened from the marine bacterium *Qs*. SW-135, which has strong substrate specificity and excellent biological properties such as alkali resistance and salt tolerance. Meanwhile, structural analysis showed that QsGH13 has a typical α/β-fold structure of the GH13 family and a special β → α loop 4 region. These studies might greatly improve our understanding of the molecular mechanism of the GH13 family, and further indicate their physiological and biochemical basis for adapting to extreme conditions, which would help to use these foundations to improve the industrial application value of α-glucosidases. Meanwhile, it has expanded the application of α-glucosidase in alcohol fermentation, glycolysis, and other industries.

## Data Availability Statement

The datasets presented in this study can be found in online repositories. The names of the repository/repositories and accession number(s) can be found below: doi: 10.2210/pdb7VOH/pdb.

## Author Contributions

JH and XZ conceived the study. XZ, KW, RJ, YZ, JL, and ZY performed the experiments and analyzed the data. XZ, XX, and JH wrote and edited its final manuscript. All authors contributed to the article and approved the submitted version.

## Conflict of Interest

The authors declare that the research was conducted in the absence of any commercial or financial relationships that could be construed as a potential conflict of interest.

## Publisher’s Note

All claims expressed in this article are solely those of the authors and do not necessarily represent those of their affiliated organizations, or those of the publisher, the editors and the reviewers. Any product that may be evaluated in this article, or claim that may be made by its manufacturer, is not guaranteed or endorsed by the publisher.

## Footnotes

1
http://www.cazy.org/

2
https://blast.ncbi.nlm.nih.gov/Blast.cgi

3
http://www.clustal.org/

4
https://espript.ibcp.fr/ESPript/ESPript/esp_tutorial.php

5
https://iupred2a.elte.hu/plot_new

6
http://g6altair.sci.hokudai.ac.jp/g6/service/pocasa/

7
http://ekhidna2.biocenter.helsinki.fi/dali/

8
https://iupred2a.elte.hu/plot_new
