# Supplementary material for: Structure and Function Insight of the α-Glucosidase QsGH13 From Qipengyuania seohaensis sp. SW-135
Source: Front Microbiol. 2022 Mar 3;13:849585. doi: 10.3389/fmicb.2022.849585 (PMC8928221; doi:10.3389/fmicb.2022.849585)
Supplement: Supplementary file 1 [file Data_Sheet_1.docx]

**Supplementary Materials**

**Table S1. Comparison of the primary characteristics of five enzymes in GH13 family.**

| **Source** | **Gene**  **names** | **PDB ID** | **Base length**  **(bp)** | **Full length**  **(aa)** | **MW**  **(Da)** | **pI** | **Charge** | **Type** | **Space Group** | Protein family |
| --- | --- | --- | --- | --- | --- | --- | --- | --- | --- | --- |
| Qipengyuania seohaensis sp. SW-135 | qsgh13 | 7VOH | 1587 | 528 | 59,299 | 4.79 | -31 | alpha-glucosidase | P2_1_2_1_2_1_ | GH13  *EC:*[*3.2.1.20*](https://enzyme.expasy.org/EC/3.2.1.20) |
| Xanthomoas campestris | xgtA | 6AAV | 1617 | 538 | 60,004 | 4.99 | -23 | alpha-glucosyltransferase | P22_1_2_1_ | GH13  *EC 2.4.1.25* |
| Halomonas sp. H11 | hag | 3WY1 | 1617 | 538 | 61,116 | 4.72 | -33 | alpha-glucosidase | P2_1_2_1_2_1_ | GH13  *EC:*[*3.2.1.20*](https://enzyme.expasy.org/EC/3.2.1.20) |
| Bacillus subtilis sp.168 | malL | 4M56 | 1687 | 561 | 66,081 | 5.15 | -23 | Oligo-1,6-glucosidase 1 | P12_1_1 | GH13  *EC:3.2.1.10* |
| [Geobacillus sp. HTA-462](https://www.rcsb.org/search?q=rcsb_entity_source_organism.taxonomy_lineage.name:Geobacillus%20sp.%20HTA-462) | gsj | 2ZE0 | 1665 | 555 | 65,177 | 5.79 | -9 | alpha-amylase | C121 | GH13  *EC:*[*3.2.1.1*](https://enzyme.expasy.org/EC/3.2.1.1) |

**Table S2. Comparison of kinetic parameters of α-glucosidase.**

| **Source** | **Type** | **pH optimum** | **Temperature optimum (°C)** | **V_max_ (U/mg)** | **K_m_ (mM)** | **References** |
| --- | --- | --- | --- | --- | --- | --- |
| *Saccharomyces cerevisiae* D-346 | Commercial enzyme | 7.0 | 30.0 | 132.00 | 0.310 | (Yamamoto et al., 2004) |
| *Aspergillus niger* | Laboratorial enzyme | 4.5 | 60.0 | 43.48 | 0.466 | (Chen et al., 2010) |
| *Thermoanaerobacter ethanolicus* | Laboratorial enzyme | 5.5 | 70.0 | 39.00 | 1.720 | (Wang et al., 2009) |
| *Pichia pastoris* | Laboratorial enzyme | 4.5 | 37.0 | 1.00 | 1.700 | (Naested et al., 2006) |
| *Qipengyuania seohaensis* sp. SW-135 | Laboratorial enzyme | 10.0 | 45.0 | 25.41 | 0.295 | This study |

**
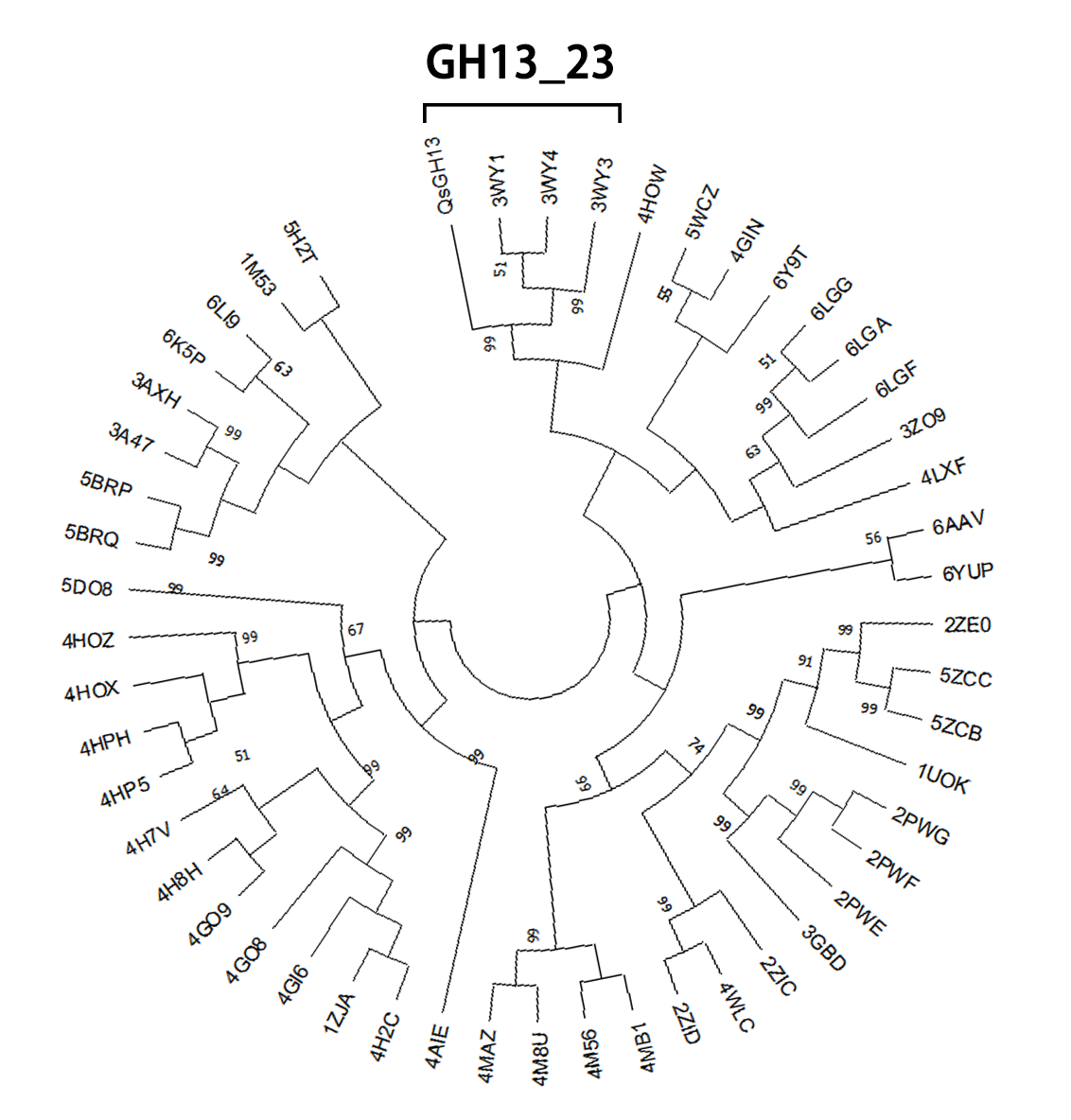
**

**Supplementary Figure 1. The adjacent phylogenetic tree sequence analysis of QsGH13.** Based on NCBI, PDB and CAZy database, the tree was constructed using MEGA software. Bootstrap values are based on 1500 replicates and only values >50% are shown. The black box represents the 23 subfamily of the GH13 family.


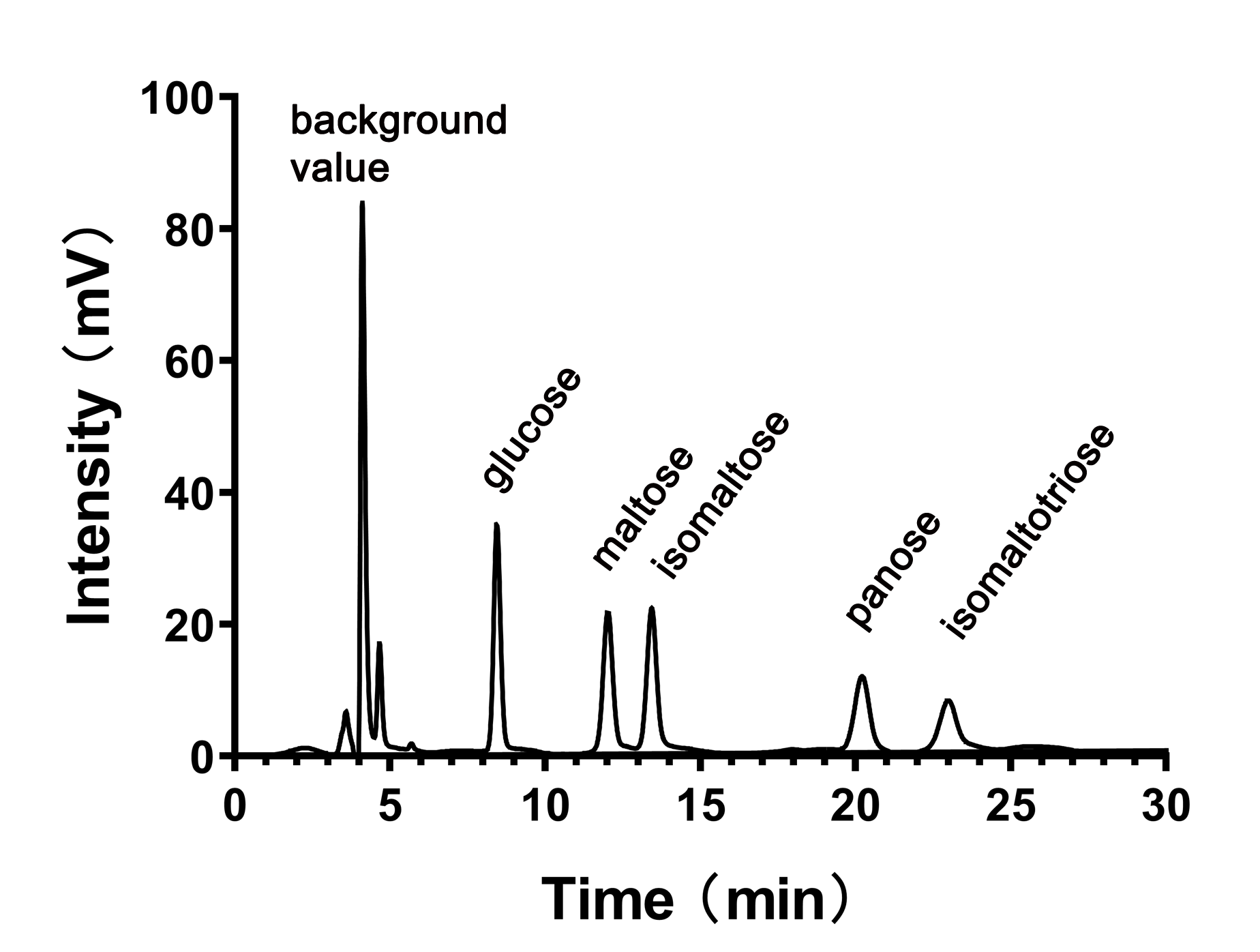


**Supplementary Figure 2. The standard samples analyzed by HPLC**. Isomaltotriose, panose, isomaltose, cellobiose and glucose came out at the peak positions of 22.99min, 20.21min, 13.43min, 12.02min and 8.44min on a Thermo Scientific™ Hypersil™ APS-2 HPLC, resrepectively.


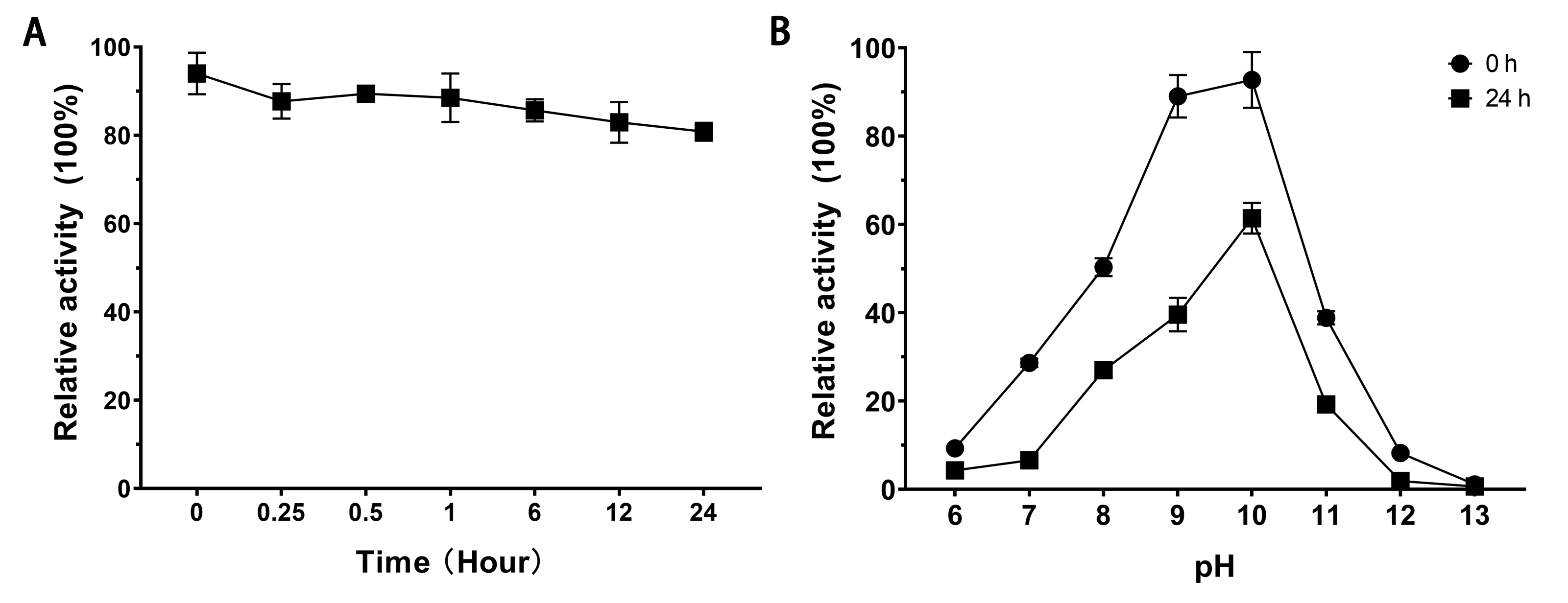


**Supplementary Figure 3. Biochemical characterization of QsGH13.** (A) The Thermostability of QsGH13 at 4℃. (B)The alkali resistance of QsGH13.


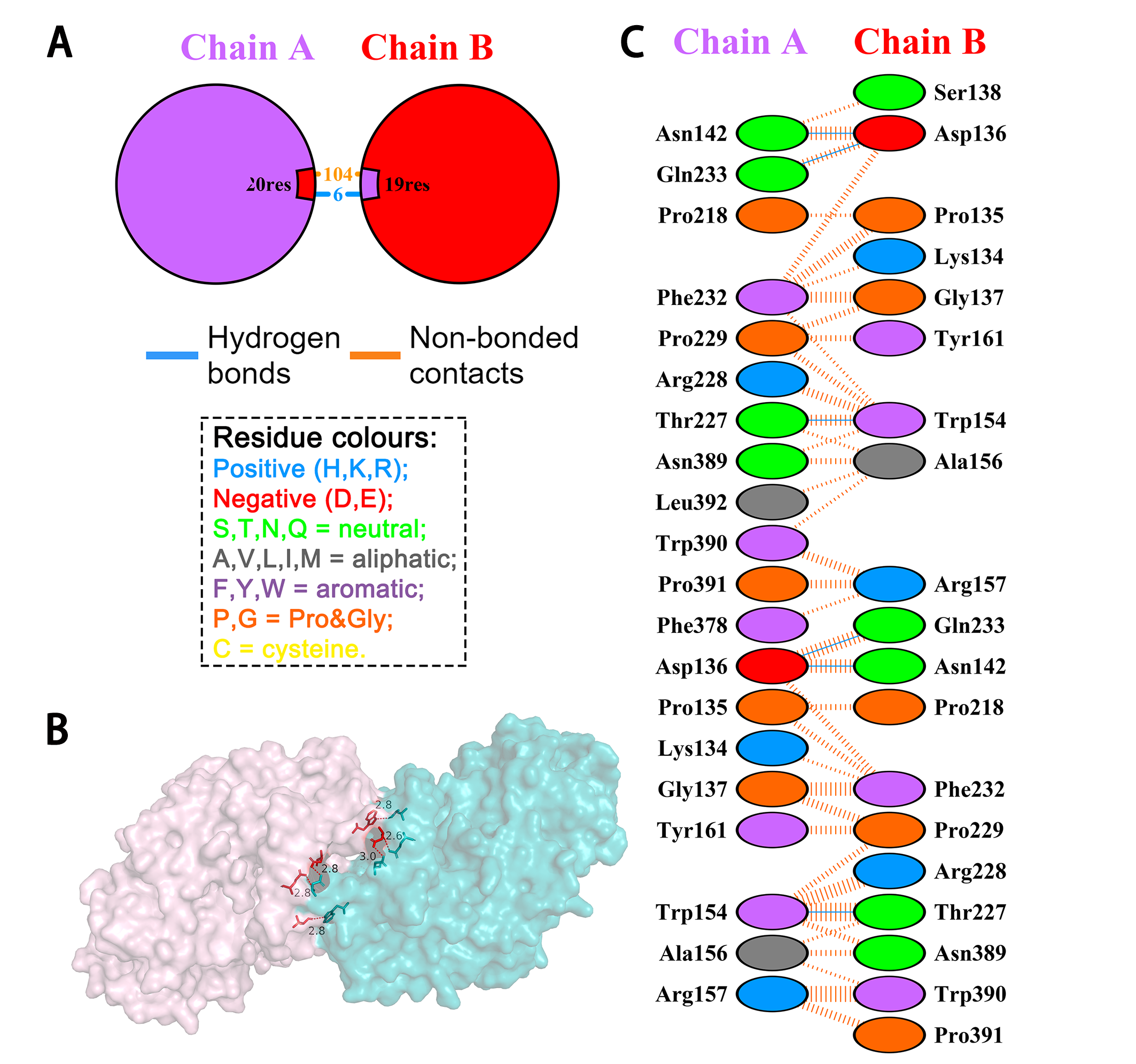


**Supplementary Figure 4. The dimeric interface in QsGH13.** (A) The monomers of QsGH13 formed dimers mainly through 6 hydrogen bonds and 104 non-bonded contacts. (B) The hydrogen-bond network between QsGH13 monomers is shown in a stick model colored with lake blue and red. The hydrogen bonds are indicated by dashed lines. (C) The residues in the dimer of QsGH13 that are involved in the formation of non-bonded contacts.


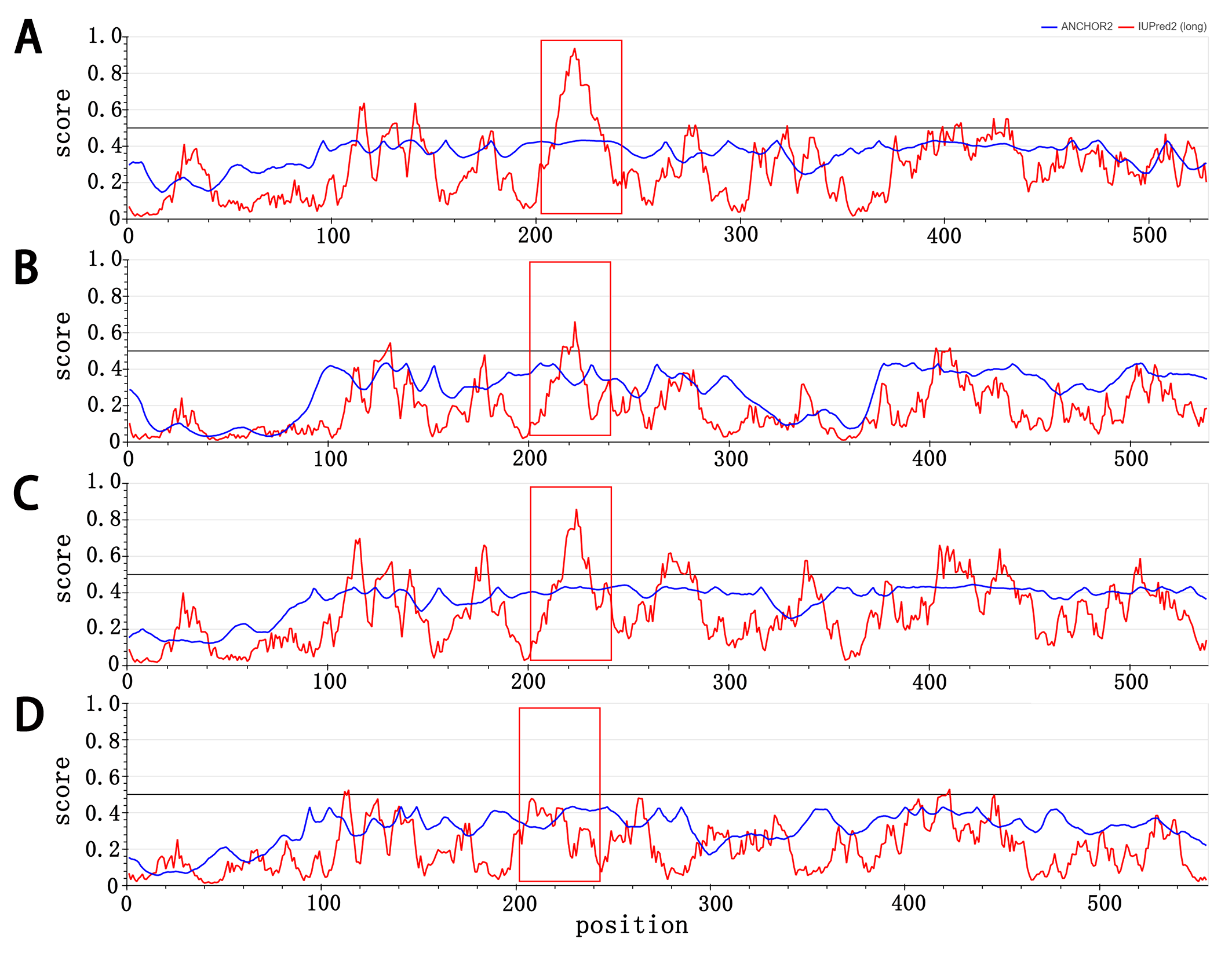


**Supplementary Figure 5. Dynamic analysis of protein.** (A) QsGH13. (B) HaG. (C) XgtA. (D) GSJ. The red box represents the β-α loop 4 region of the protein.


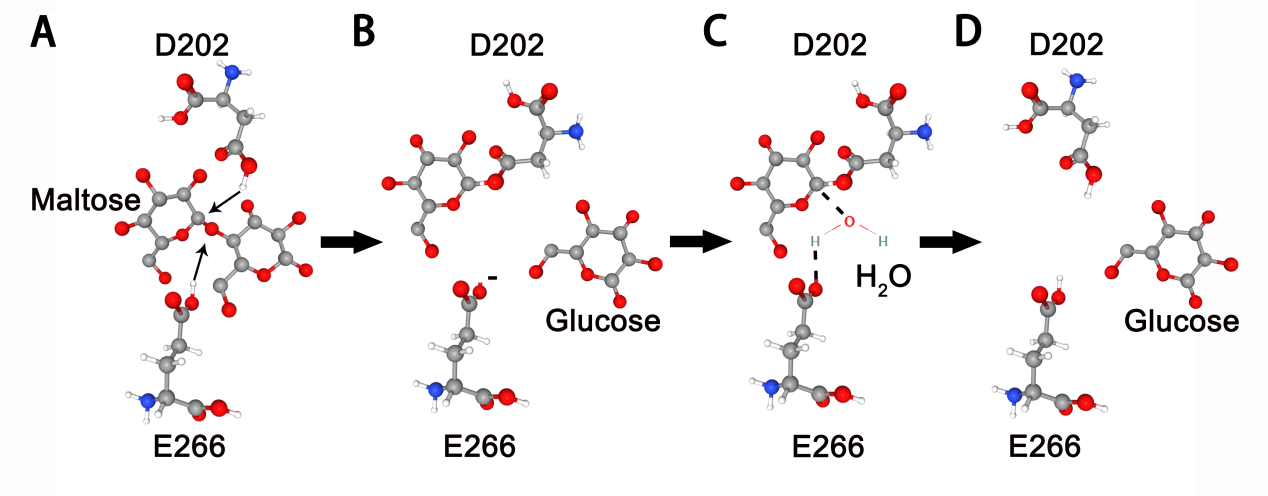


**Supplementary Figure 6. The mechanism of QsGH13 hydrolyzing maltose.**
